# Diagnostic Accuracy and Clinical Utility of Salivary Biomarkers in Oral Squamous Cell Carcinoma: A Meta-Analysis

**DOI:** 10.3390/cancers18060970

**Published:** 2026-03-17

**Authors:** Arbi Wijaya, Vera Julia, Nurtami Soedarsono, Lilies D. Sulistyani, Moh Adhitya Latief, Turmidzi Fath, Bayu Brahma, Alif Rizqy Soeratman, Denni Joko Purwanto, Yutaro Higashi, Tsuyoshi Sugiura

**Affiliations:** 1 Department of Oral & Maxillofacial Surgery, Faculty of Dentistry, Universitas Indonesia, Jakarta 10430, Indonesia; arbi.wijaya.t2@dc.tohoku.ac.jp (A.W.);; 2 Division of Oral and Maxillofacial Oncology and Surgical Sciences, Graduate School of Dentistry, Tohoku University, Sendai 980-0872, Miyagi, Japan; 3 Department of Oral Biology, Faculty of Dentistry, Universitas Indonesia, Jakarta 10430, Indonesia; 4 Division of Surgical Oncology, Dharmais National Cancer Center Hospital, Jakarta 11420, Indonesia

**Keywords:** oral squamous cell carcinoma, saliva, biomarkers, diagnostic accuracy, sensitivity, specificity

## Abstract

**Background:** Oral squamous cell carcinoma (OSCC) remains a major global health burden due to delayed diagnosis. Although salivary biomarkers have been explored in previous meta-analyses, these studies were limited to specific biomarker types. **Methods:** This study followed PRISMA guidelines and was registered in PROSPERO (CRD 420261296936). PubMed, Scopus, MEDLINE, and CINAHL were searched for diagnostic accuracy studies of salivary biomarkers for OSCC. Studies providing sufficient data to construct 2 × 2 tables were included. Pooled sensitivity, specificity, DOR, and HSROC curves were estimated using a bivariate random-effects model, and study quality was assessed using QUADAS-2. **Results:** Eighteen studies comprising 1647 participants yielded 45 diagnostic datasets across multiple biomarker classes. The pooled sensitivity and specificity were 0.64 (95% CI: 0.59–0.69) and 0.71 (95% CI: 0.66–0.76), respectively. The pooled DOR was 4.53 (95% CI: 3.18–6.47), indicating moderate discriminatory ability, with an AUC of 0.75 (95% CI: 0.71–0.79). Fagan’s nomogram analysis demonstrated that these biomarkers are not suitable for screening the general population and should be reserved for enriched populations (pre-test probability > 10%). **Conclusions:** Salivary biomarkers demonstrate moderate but highly heterogeneous diagnostic accuracy. Clinical utility is context-dependent and limited to enriched populations with a baseline probability of OSCC >10%. In screening the general population (prevalence < 0.01%), these tests offer no significant clinical utility. They should be considered complementary triage tools rather than definitive diagnostic modalities.

## Simple Summary

Salivary biomarkers demonstrate moderate diagnostic accuracy and limited clinical utility when applied as complementary tools for OSCC classification in high-prevalence settings where the pre-test probability exceeds 10%. Their overall performance supports a role in their use as risk-stratification instruments in carefully selected patient populations, rather than as stand-alone diagnostics or screening tests for the general population. Notably, these biomarkers exhibit greater detection capacity. The test has greater inclusion than exclusion, meaning it is more effective at identifying potentially positive cases than at reliably ruling out disease. As a consequence, capacity and post-test probabilities remain clinically concerning even after a negative result, particularly in high-risk individuals and high-risk patients.

## 1. Introduction

According to the Global Cancer Observatory (GLOBOCAN) 2020 data, oral squamous cell carcinoma (OSCC) accounts for over 90% of oral malignancies, representing a formidable global health burden with high morbidity and mortality [1,2,3]. Despite advances in therapeutic modalities, OSCC remains a leading cause of cancer-related mortality in the head and neck region [4,5]. The etiology is predominantly multifactorial, driven by lifestyle factors such as tobacco and alcohol use, betel quid chewing, and viral infections, along with systemic contributors, including low socioeconomic status and limited health literacy [6,7].

The prognosis of OSCC is critically stage dependent; while the five-year survival rate exceeds 80% for early-stage disease (Stage I–II), it declines to approximately 60% for advanced-stage (Stage III–IV) presentations [8]. Although early detection enables curative intervention and significantly improves survival, most cases are diagnosed at advanced stages, resulting in diminished therapeutic efficacy [9]. Furthermore, conventional tissue biopsy, the diagnostic gold standard, is inherently invasive and provides only a localized, cross-sectional snapshot of tumor biology, often failing to capture the malignancy’s spatial heterogeneity and dynamic molecular evolution [10]. These diagnostic limitations highlight the urgent need for minimally invasive screening tools capable of early-stage detection [10].

Saliva has emerged as a promising diagnostic biofluid, offering a non-invasive, cost-effective, and repeatable alternative to liquid biopsies [11]. Recent advances in “omics” technologies, including proteomics, genomics, and transcriptomics, have identified a diverse array of salivary biomarkers, including cytokines, cell-free DNA, and microRNAs, that reflect the molecular landscape of the tumor microenvironment [12]. Because saliva integrates biological signals from local tumor tissues, the systemic circulation, and the oral microbiome, it provides a unique diagnostic window into disease pathophysiology [13]. However, existing reviews largely assess isolated biomarkers, without integrated evaluation of diagnostic performance or clinical utility [14,15,16]. Consequently, the comparative diagnostic utility across different biomarker classes remains poorly defined. This systematic review and meta-analysis provide a comprehensive evaluation of salivary biomarkers for OSCC detection, synthesizing global evidence across diverse molecular targets and analytical platforms to establish their collective clinical utility.

## 2. Materials and Methods

### 2.1. Study Protocol

**Figure 1:**
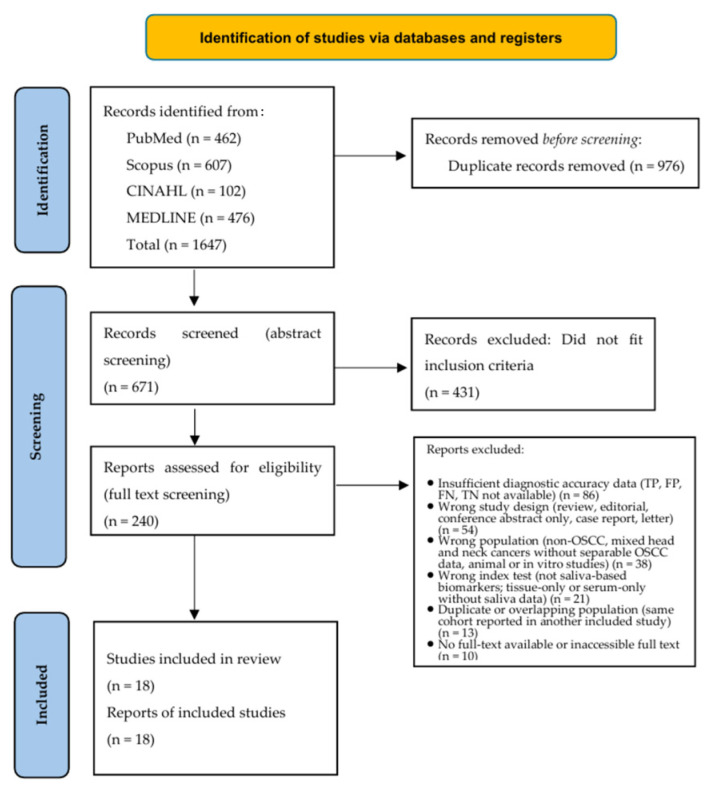
PRISMA flowchart of database searching and study selection.

This systematic review protocol was registered in the International Prospective Register of Systematic Reviews (PROSPERO) under the number CRD420261296936. The meta-analysis was conducted in accordance with the Preferred Reporting Items for Systematic Reviews and Meta-Analysis (PRISMA) guidelines (Figure 1).

### 2.2. Search Strategy

A systematic literature search was conducted independently by two investigators (A.W. and N.S.) across four primary electronic databases: PubMed, Scopus, CINAHL, and MEDLINE. The search strategy employed database-specific Boolean strings:

“(oral squamous cell carcinoma OR OSCC OR oral cancer) AND (saliva OR salivary OR oral fluid) AND (miRNA OR microRNA OR non-coding RNA OR cfDNA OR cell-free DNA OR ctDNA OR circulating tumor DNA OR DNA methylation) OR (protein OR proteomic) OR (cytokine OR interleukin OR chemokine) OR (metabolomic OR metabolite) AND (diagnosis OR diagnostic accuracy OR sensitivity OR specificity OR ROC OR AUC)”.

All identified records were imported into the Rayyan QCRI platform (https://www.rayyan.ai/, accessed on 27 July 2025) for efficient duplicate removal and blinded screening. In the first phase, two reviewers (A.W. and N.S.) independently screened titles and abstracts against predefined eligibility criteria. Subsequently, a comprehensive full-text review was conducted to select studies that explicitly reported raw diagnostic metrics or provided sufficient data to reconstruct them. Discrepancies at any stage were resolved through iterative discussion and consensus; when a resolution could not be reached, a third senior reviewer (V.J.) adjudicated the final inclusion.

### 2.3. Eligibility Criteria

Study eligibility was defined using the PICOS framework, with stringent criteria to ensure diagnostic consistency across diverse biomarker modalities and clinical cohorts. We included diagnostic accuracy studies comprising case–control, cross-sectional, and cohort designs that enrolled patients with Oral Squamous Cell Carcinoma (OSCC) and healthy control cohorts specifically screened to exclude confounding oral inflammatory pathologies. The index tests encompassed salivary biomarkers across three molecular strata, proteomic, genomic, and transcriptomic, all of which required validation using standardized molecular or immunoassay techniques to ensure analytical reproducibility. To minimize misclassification bias and provide a uniform benchmark for accuracy, the reference standard was strictly limited to histopathological confirmation, the definitive clinical gold standard. Furthermore, to maintain the integrity of the statistical synthesis, studies were required to provide granular raw data, specifically true positives (TP), false positives (FP), true negatives (TN), and false negatives (FN), enabling reconstruction of 2 × 2 contingency tables for robust calculation of pooled sensitivity, specificity, and the area under the receiver operating characteristic curve (AUC).

### 2.4. Data Extraction and Quality Assessment

Data from eligible studies were extracted into a standardized form, capturing study metadata (author, year, country, and design), clinico-pathological characteristics (sample size, age, sex, smoking history, and systemic comorbidities), and technical parameters (biomarker type, detection platform, and normalization methods). For the meta-analysis, raw diagnostic metrics (true positives [TP], false positives [FP], false negatives [FN], and true negatives [TN]) and sensitivity, specificity, and the Area Under the Curve (AUC) were recorded.

The risk of bias analysis of the included studies was independently appraised by two investigators (A.W. and N.S.) using the Quality Assessment of Diagnostic Accuracy Studies-2 (QUADAS-2) tool. To ensure transparency and minimize subjectivity, each study was evaluated across four essential domains: patient selection, index test, reference standard, and flow and timing. In the patient selection domain, we specifically scrutinized the recruitment strategy to differentiate between consecutive/random “diagnostic-intent” cohorts (low risk) and case–control “two-gate” designs (high risk), the latter of which can lead to overestimations of diagnostic accuracy due to spectrum bias. Within the index test domain, we assessed whether diagnostic thresholds were pre-specified and whether laboratory personnel remained blinded to the reference standard results to prevent review bias. For the reference standard, rigor was ensured by confirming all diagnoses via histopathology, thereby eliminating misclassification. The flow and timing domain addressed potential attrition bias by evaluating the interval between saliva collection and biopsy and by including all recruited participants in the final analysis. Inter-reviewer discrepancies in scoring were resolved through rigorous consensus-building sessions or, where necessary, via adjudication by a third senior investigator (V.J.), ensuring a high degree of inter-rater reliability and objective appraisal.

### 2.5. Statistical Analysis

Statistical analysis was conducted using Stata 19.0 (StataCorp LLC, College Station, TX, USA) via the midas and metandi modules. We used a bivariate random-effects model to calculate pooled sensitivity, specificity, and Diagnostic Odds Ratio (DOR) with 95% confidence intervals (CIs). Global discriminatory capacity was quantified using the Area Under the Curve (AUC) and a Summary Receiver Operating Characteristic (SROC) model. Statistical heterogeneity was evaluated using the Cochran Q test and the I^2^ statistic, with inter-variable correlation heatmaps used to distinguish between threshold and spectrum effects. Clinical utility was assessed using Fagan’s nomograms to determine post-test probabilities. Potential publication bias was examined using Deeks’ funnel plot asymmetry test, with statistical significance defined as *p* < 0.05.

## 3. Results

### 3.1. Study Selection

The systematic search yielded 1647 records across PubMed (n = 462), Scopus (n = 607), MEDLINE (n = 476), and CINAHL (n = 102). After removing 976 duplicates, 671 unique records underwent title and abstract screening, resulting in the exclusion of 431 citations. Full-text evaluation was performed for 240 potentially eligible articles. Of these, 222 reports were excluded based on the following criteria: insufficient diagnostic performance metrics (TP, FP, FN, TN; n = 86), non-primary research designs (n = 54), heterogeneous patient populations (n = 38), non-salivary index tests (n = 21), overlapping cohorts (n = 13), and inaccessible full texts (n = 10). Ultimately, 18 studies comprising 1628 participants (OSCC cases and controls) met all eligibility criteria for inclusion in the meta-analysis. The full extracted dataset is presented in Table S1. The selection process is detailed in the PRISMA flow diagram (Figure 1) and the PRISMA checklist is provided in Table S2.

### 3.2. Study Characteristic

As summarized in Table 1, most studies used a case–control design and evaluated a broad range of biomarkers, including protein-based markers, cytokines, cell-free DNA integrity indices, single and panel-based microRNAs, circular RNAs, and extracellular vesicle-derived biomarkers, using platforms such as ELISA, RT–qPCR, microarray, NGS, and proteomic approaches. Baseline demographic and clinical characteristics of OSCC patients and control groups are detailed in Table 2, demonstrating heterogeneity in age distribution, sex ratio, smoking status, and control definitions, with variable matching strategies across studies. Diagnostic performance metrics extracted from each study are summarized in Table 3, showing wide variability in TP, FP, TN, FN, sensitivity, specificity, and AUC across biomarker type.

### 3.3. Sensitivity and Specificity

**Figure 2:**
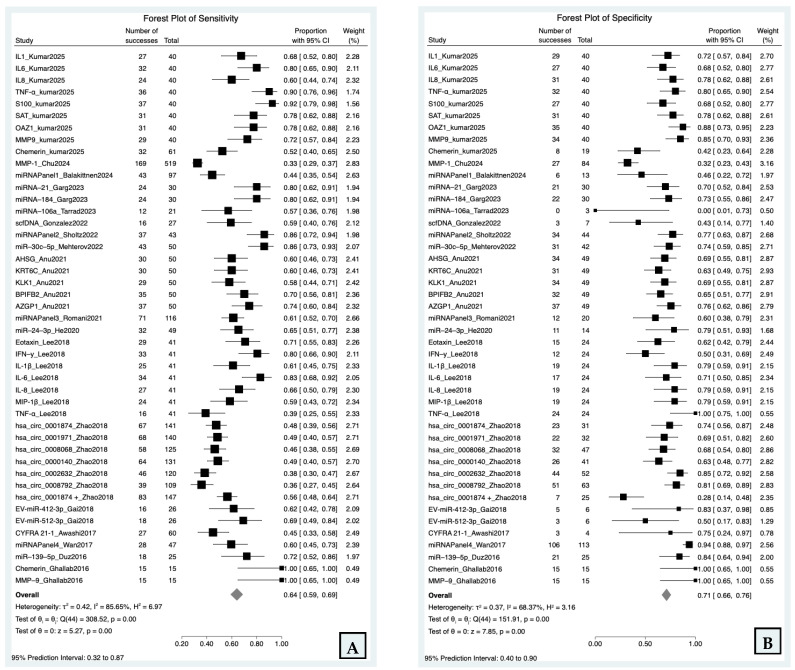
Forest plots of pooled sensitivity (**A**) and specificity (**B**) of salivary biomarkers for oral squamous cell carcinoma, with 95% confidence intervals and bivariate random-effects pooled estimate.

The forest plots (Figure 2) summarize the diagnostic accuracy of salivary biomarkers across the 45 included datasets. Using a bivariate random-effects model, the meta-analysis yielded a pooled sensitivity of 0.64 (95% CI: 0.59–0.69) and a pooled specificity of 0.71 (95% CI: 0.66–0.76). Extreme heterogeneity was observed for both primary diagnostic indices. The pooled sensitivity had an I^2^ of 85.65% and *p* = 0.00, while the pooled specificity had an I^2^ of 68.37% and *p* = 0.00. The 95% prediction interval suggests that sensitivity in future studies could range from 32% to 87% and specificity 40% to 90% from reflecting extreme between-study heterogeneity.

### 3.4. Receiver Operating Characteristic (SROC) Curve

**Figure 3:**
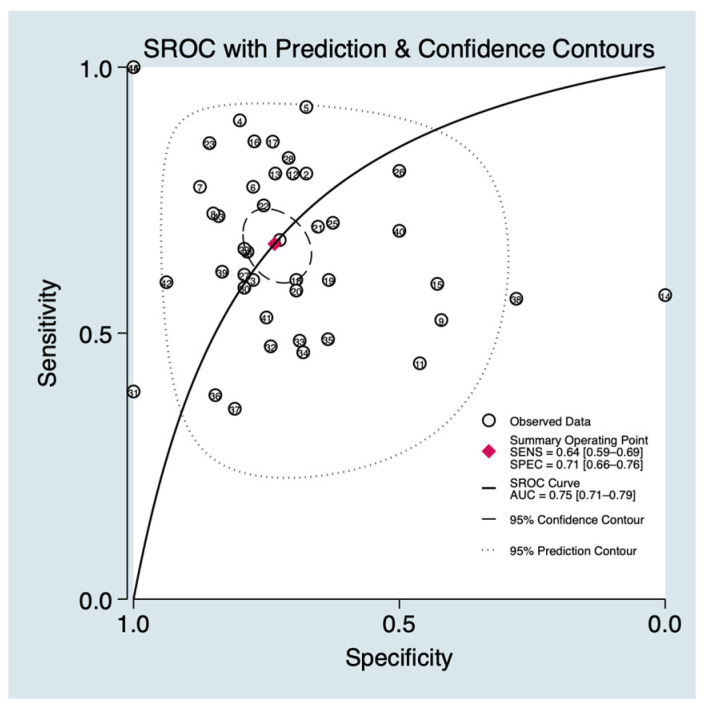
Summary Receiver Operating Characteristic (SROC) curve depicting the overall diagnostic performance of salivary biomarkers.

The global diagnostic performance of the evaluated salivary biomarkers was summarized using the Summary Receiver Operating Characteristic (SROC) curve (Figure 3) to account for potential threshold effects and provide a comprehensive measure of discriminatory capacity. The analysis yielded a pooled Area Under the Curve (AUC) of 0.75 (95% CI: 0.71–0.79), indicating moderate overall accuracy in distinguishing OSCC cases from healthy control.

### 3.5. Global Discriminatory Capacity via Pooled Diagnostic Odds Ratio (DOR)

**Figure 4:**
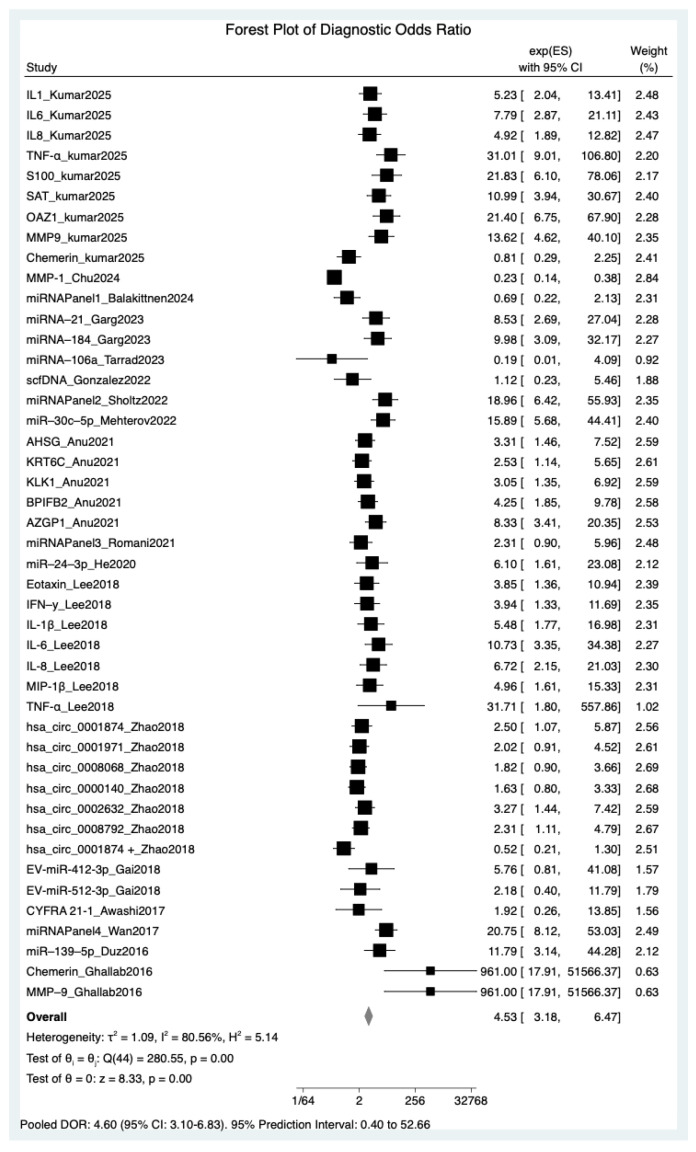
Forest plot of pooled Diagnostic Odds Ratios (DORs) summarizing the global discriminatory capacity.

The global diagnostic performance of salivary biomarkers was quantified using the Diagnostic Odds Ratio (DOR) (Figure 4). Based on a random-effects meta-analysis of 45 datasets, the pooled DOR was 4.53 (95% CI: 3.18–6.47). This indicates that the odds of a positive salivary biomarker result are 4.6 times higher in patients with OSCC than in healthy controls. Extreme statistical heterogeneity was confirmed (I^2^ = 80.56%; *p* < 0.001), underscoring that although salivary biomarkers are effective for OSCC risk stratification, their performance depends on the specific molecular targets and clinical cohorts evaluated.

### 3.6. Deek’s Funnel Plot Publication Bias

**Figure 5:**
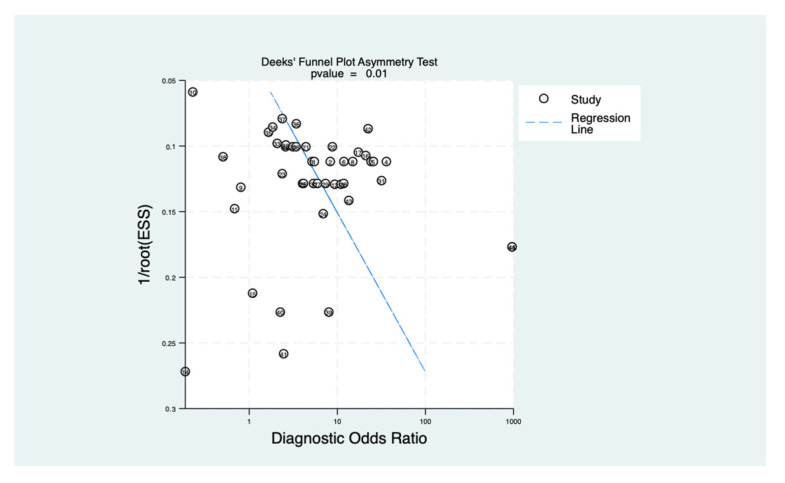
Deeks’ funnel plot assessing potential publication bias and small-study effects.

Potential publication bias and small-study effects were evaluated using Deeks’ funnel plot asymmetry test (Figure 5). Linear regression of the diagnostic log odds-ratio (lnDOR) against the inverse square root of the effective sample size (1√ESS) yielded a positive slope, reflecting some scatter among lower-precision studies. However, the relatively balanced distribution of studies around the regression line suggests the absence of systematic bias. The statistical significance of this asymmetry, as determined by the regression test (*p*-value = 0.001), indicates a lack of publication bias, confirming that the pooled diagnostic estimates for salivary biomarkers are robust and not substantially skewed by small-study effects. However, with a moderate number of studies (n = 18) and extremely high heterogeneity (I^2^ = 80.56%), this test has limited statistical power to detect bias. The absence of statistical significance should not be interpreted as definitive evidence of the absence of bias.

### 3.7. Clinical Utility via Fagan’s Nomogram Analysis

**Figure 6:**
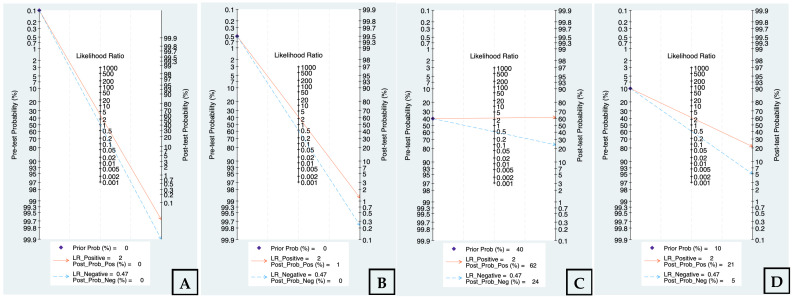
Analysis of Fagan’s nomogram in different clinical prevalence scenarios (0.01% (**A**), 0.5% (**B**), 10% (**C**), and 40% (**D**)).

To assess clinical applicability across practice settings, Fagan’s nomogram was applied using the pooled likelihood ratios (LR+ = 2; LR− = 0.47) under four pre-test probability scenarios (0.01%, 0.5%, 10%, and 40%) (Figure 6 and Table 4). In very-low-prevalence settings (0.01%), a positive result yielded a negligible post-test probability (0%), indicating limited value for population-level screening. At a low pre-test probability of 0.5%, a positive result increased the post-test probability to approximately 1%, whereas a negative result reduced it to 0%, supporting a potential role as a triage tool rather than a definitive diagnostic test. In clinically enriched settings (pre-test probability 10%), a positive result increased the post-test probability to 21%, while a negative result lowered it to 5%, suggesting moderate rule-in and partial rule-out utility. In high-suspicion/referral scenarios (pre-test probability 40%), post-test probabilities were 62% following a positive result and 24% following a negative result, demonstrating that clinical impact becomes more meaningful as baseline risk increases.

### 3.8. Assessment of Inter-Variable Correlation and Threshold Effects via Heatmap Analysis

**Figure 7:**
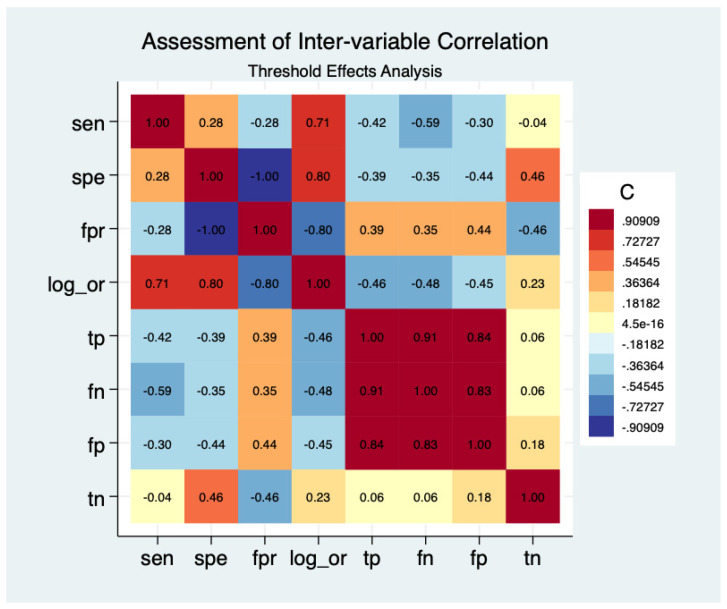
Correlation heatmap illustrating inter-variable relationships among diagnostic parameters and assessing potential threshold effects.

The inter-variable correlation matrix (Figure 7) was used to assess internal consistency and potential systematic biases in the diagnostic parameters. The weak positive correlation between sensitivity and specificity (r = 0.28) suggests heterogeneity in the intrinsic quality of the biomarkers, rather than threshold effects, since some biomarkers perform better than others in both metrics. Subsequently, strong positive associations between the Log Diagnostic Odds Ratio (lnDOR) and both sensitivity (r = 0.71) and specificity (r = 0.80) further confirm the mathematical stability of the bivariate model. These findings indicate that the extreme heterogeneity observed is driven by intrinsic biological and methodological diversity across cohorts rather than systemic variations in diagnostic cut-off points.

### 3.9. Investigation of the Sources of Heterogeneity

#### 3.9.1. Subgroup Analysis

To address the extreme heterogeneity observed in this study (I^2^ for sensitivity = 86.65% and specificity = 68.37%), we performed subgroup analyses across five parameters: biomarker type, biomarker composition (individual vs. panel), detection platform, geographic region, and study quality (Figure S1). No significant differences in diagnostic performance were observed when stratifying by biomarker type (*p* = 0.97), individual vs. panel composition (*p* = 0.90), detection platform (*p* = 0.21), or geographic region (*p* = 0.80), as extreme residual heterogeneity (I^2^ > 75%) persisted across nearly all strata. However, study quality (QUADAS-2) was identified as a significant source of between-study variance (Qb (1) = 8.42; *p* = 0.004), with studies classified as “High Risk” of bias yielding a significantly higher pooled log odds-ratio (1.71 [95% CI: 1.38, 2.05]) than those with “Some Concerns” (−0.38 [95% CI: −1.76, 0.99]). These findings indicate that while the observed inconsistency is likely multifactorial, stemming from biological diversity and technical variations, methodological quality is a primary driver of the variance, necessitating that the overall pooled estimates be interpreted with extreme caution, as they represent a broad average of highly disparate primary data.

#### 3.9.2. Univariable Meta-Regression Findings

**Figure 8:**
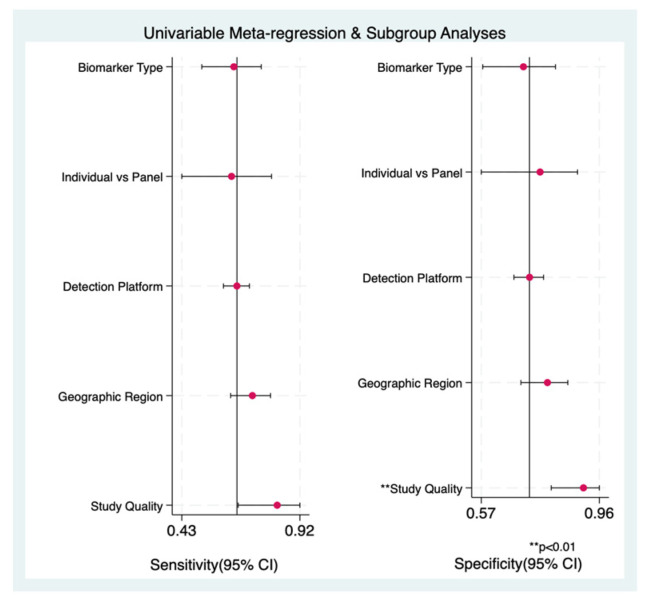
Univariable meta-regression and subgroup analysis to investigate source of heterogeneity.

Univariable meta-regression confirmed that study quality (QUADAS-2 score) was the primary and most significant source of heterogeneity in this study (Figure 8). Methodological quality significantly influenced both sensitivity (*p* = 0.05) and specificity (*p* = 0.01), with the joint model yielding a *p*-value of 0.01. Specifically, studies with high risk of bias demonstrated significantly higher diagnostic accuracy (Sensitivity: 0.82 [0.66–0.92]; Specificity: 0.91 [0.80–0.96]) than the overall pooled estimate. This suggests that variations in patient selection or in the application of the reference standard are major contributors to the observed variance.

#### 3.9.3. Leave-One-Out Sensitivity

**Figure 9:**
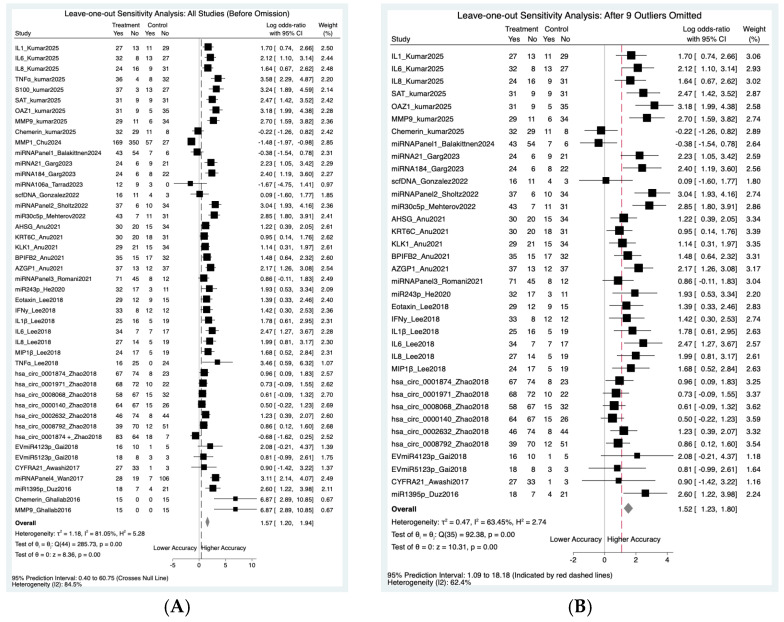
Leave-one-out sensitivity analysis: (**A**) before omission and (**B**) after omission of potential outlier.

To assess the stability of the diagnostic estimates, we performed a leave-one-out sensitivity analysis (Figure 9). Initially, extreme heterogeneity (I^2^ = 81.05%) and a wide 95% prediction interval (PI: 0.40–60.75) that crossed the null line necessitated extreme caution in interpreting the pooled results. However, the systematic omission of nine influential outliers; MMP1_Chu2024, miRNAPanel4_Wan2017, hsa_circ_0001874_Zhao2018, TNFα_kumar2025, S100_kumar2025, miRNA106A_Tarrad2023, TNFα_Lee2018, Chemerin_Ghallab2016, and MMP9_Ghallab2016 significantly reduced the I^2^ to 63.45%. Following this adjustment, the 95% prediction interval narrowed to 1.09 to 18.18, no longer crossing the null line.

### 3.10. Risk of Bias Assessment Results

**Figure 10:**
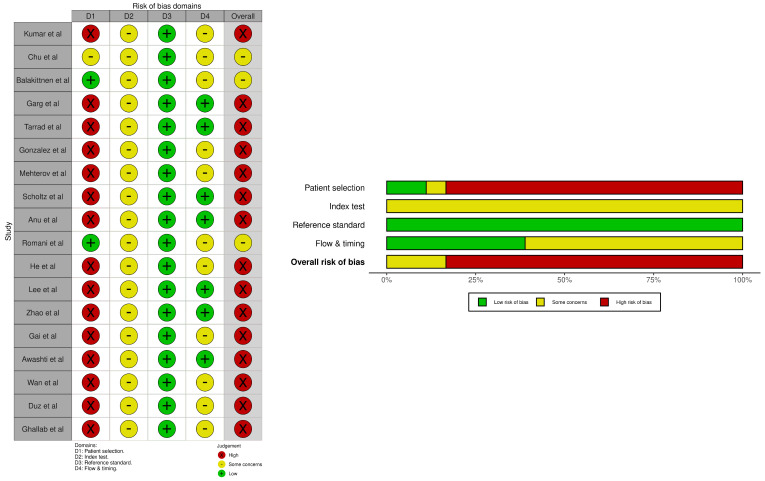
Quality assessment included studies using the Quality Assessment of Diagnostic Accuracy Studies (QUADAS-2).

The methodological quality appraisal using the QUADAS-2 tool revealed a high risk of bias in patient selection across the majority of the 18 included studies, primarily due to the use of case–control (two-gate) designs that compared known OSCC patients with healthy controls rather than using consecutive “diagnostic-intent” cohorts (Figure 10). Furthermore, the index test domain was characterized by “some concerns” (yellow) throughout, likely stemming from a lack of pre-specified diagnostic thresholds or unclear blinding of laboratory personnel to the reference results. Conversely, the reference standard domain exhibited a consistently low risk of bias (green), as all studies employed histopathological confirmation, the clinical gold standard for definitive diagnosis. The flow and timing domain demonstrated mixed results, with some concerns arising from unclear reporting of the interval between saliva collection and biopsy, as well as the exclusion of specific participants from the final analysis.

## 4. Discussion

This meta-analysis provides a comprehensive evaluation of the diagnostic landscape for salivary biomarkers in oral squamous cell carcinoma (OSCC), addressing not only diagnostic accuracy but also clinical utility. Results of our study, reporting a pooled sensitivity of 0.64 (95% CI: 0.59–0.69), pooled specificity of 0.71 (95% CI: 0.66–0.76), and 0.75 (95% CI: 0.71–0.79), indicate that while salivary diagnostics offer a balanced diagnostic profile, their accuracy remains moderate relative to traditional clinical benchmarks. The calculated Diagnostic Odds Ratio (DOR) of 4.6 further supports the moderate discriminatory capacity, indicating that the odds of a positive salivary biomarker result are approximately 4.6 times higher in patients with OSCC than in healthy controls. However, these summary metrics alone do not fully elucidate the practical utility of saliva in a frontline clinical setting [35].

To bridge the gap between statistical findings and clinical decision-making, Fagan’s nomogram (based on Bayes’ theorem) was used to contextualize these results in terms of diagnostic probabilities [36]. Our analysis using Fagan’s nomogram demonstrates that the clinical utility of these salivary biomarkers is strictly context-dependent and highly contingent on disease prevalence within the target population. Based on sensitivity analyses across a range of prevalence scenarios (0.01% to 40%), these biomarkers are clearly unsuitable for general population screening; in settings where the pre-test probability is below 1%, a positive test result yields a post-test probability of ≤1%, rendering the test clinically meaningless for mass screening. Consequently, the application of these salivary signatures should be reserved for clinically enriched populations or high-risk cohorts where the pre-test probability exceeds 10%. In such symptomatic or referral-based settings (10–40% prevalence), a positive result effectively doubles or triples the likelihood of disease, shifting the probability to 21–62%, thereby establishing these biomarkers as valuable adjunctive tools for clinical triage and targeted diagnostic workups. As noted by Speight et al., the clinical value of a screening tool is predicated on its ability to minimize false negatives in high-prevalence populations [37]. This inherent asymmetry indicates that salivary biomarkers currently function more effectively as risk-stratification or “rule-in” tools than as definitive exclusionary tests [38]. Thus, we propose that salivary biomarkers should be utilized as adjunctive stratification tools rather than definitive diagnostic modalities.

Salivary diagnostics offer a compelling advantage by potentially mitigating the limitations of OSCC spatial heterogeneity [36]. While localized biopsy may fail to capture minor tumor clones or aggressive subregions, saliva, as a “pooled” biofluid, can offer a more global molecular snapshot of the oral cavity [37]. This is particularly relevant given the concept of “field cancerization,” originally described by Slaughter et al. [39]. Saliva represents a unique reservoir of molecular signals, such as early epigenetic silencing and proteomic alterations, that can emerge in clinically normal-appearing mucosa before any visible structural changes develop [38].

Among biomarkers with adequate sample sizes (n > 100), miRNA panels and MMP-1 showed the most consistent performance across studies, with sensitivity ranging from 85% to 90% and specificity from 78% to 90% [18,19]. Although MMP-9 and chemerin showed perfect discrimination (100%/100%) in a small study (n = 30, Ghallab 2016), this apparent performance should be interpreted cautiously [34]. Perfect performance in small case–control studies often reflects overfitting and optimism bias rather than true diagnostic ability. These results have not been replicated in larger cohorts and should be interpreted with extreme caution. More credible evidence comes from larger studies reporting sensitivities of 86–93% and specificities of 78–90% (n = 110–367 participants) [18,19]. MMP-9 and chemerin require rigorous validation in prospective studies with adequate statistical power before clinical consideration.

The present meta-analysis is subject to several important limitations. The high heterogeneity observed indicates that variations in salivary biomarker types substantially influence diagnostic outcomes. The predominance of case–control designs may overestimate accuracy relative to real-world clinical populations with heterogeneous oral lesions, and the lack of standardized cut-off values remains a major barrier to implementation. In addition, small-sample studies reporting near-perfect or 100% diagnostic performance should be interpreted with caution, as such findings may reflect overfitting or optimism bias rather than true clinical validity; emphasizing these results without appropriate caveats is scientifically problematic.

Future research should therefore transition toward large-scale, multi-center prospective trials that focus on “diagnostic-intent” populations rather than healthy controls. Prioritizing the development of multi-omic panels integrating proteomic, transcriptomic, and epigenetic markers may provide the synergistic sensitivity needed to meet the diagnostic threshold. Moreover, integrating artificial intelligence and machine-learning algorithms to interpret complex salivary signatures represents a promising avenue for personalized oral cancer screening [40]. In light of evidence indicating that lactate and lactylation are pivotal mediators linking tumor metabolism and epigenetic regulation, future research should incorporate these metabolic signatures into multi-omic panels as novel OSCC biomarkers to enhance the sensitivity and biological depth of personalized salivary screening [41]. Furthermore, resolving the extremely high heterogeneity identified in current diagnostic evidence, which our analysis primarily attributed to methodological quality, will require the adoption of standardized protocols (such as the QUADAS-2 framework) and the investigation of more stable metabolic indicators to ensure that future pooled estimates provide a consistent and valid representation of real-world clinical scenarios.

## 5. Conclusions

Salivary biomarkers demonstrate moderate but highly heterogeneous diagnostic accuracy (sensitivity 0.63, I^2^ = 91.88%; specificity 0.70, I^2^ = 82.13%). Clinical utility is context-dependent and limited to enriched populations with a baseline probability of OSCC >10%. In screening the general population (prevalence < 0.1%), these tests offer no significant clinical utility. They should be considered complementary triage tools rather than definitive diagnostic modalities.

## Figures and Tables

**Table 1:** Characteristics of included studies.

First Author	Year	Country	Study Design	Sample Size			Biomarker Evaluated	Detection Platform	Normalization/Internal Control
				OSCC	Control	Total			
Kumar et al. [17]	2025	India	Case Control	40	40	80	IL1	RT–qPCR	RT-PCR housekeeping/reference gene: B2M
							IL6		
							IL8		
							TNF-α		
							S100P		
							SAT		
							OAZ1		
							MMP9		
							Chemerin		
Chu et al. [18]	2024	Taiwan	Cross-sectional/Nested Case Control	196	171	367	MMP-1	MMP-1 by rapid strip test (RST), reporting relative light units (RLU)	Control line (C-line) to verify a valid test (RLU > 100)
Balakittnen et al. [19]	2024	Australia	Prospective Cohort	50	60	110	Panel: miR–7–5p, miR–10b–5p, miR–182–5p, miR–215–5p, miR–431–5p, miR–486–3p, miR–3614–5p, and miR–4707–3p	NGS, RT–qPCR	miR–191–5p, miR–484, and SNORD96A
Garg et al. [20]	2023	India	Case Control	30	30	60	miRNA–21	RT–qPCR	U6 snRNA
							miRNA–184		
Tarrad et al. [21]	2023	Egypt	Case Control	12	12	24	miRNA–106a	RT–qPCR	SNORD–68
Rapado-González et al. [22]	2022	Spain	Case Control	19	15	34	scfDNA integrity indexes • ALU115/ALU60 • ALU247/ALU60	RT–qPCR	ALU60 amplicon
Mehterov et al. [23]	2022	Bulgaria	Case Control	34	12	45	miR–30c–5p	RT–qPCR	RNU6 and SNORD72
Scholtz et al. [24]	2022	Hungary	Case Control	43	44	87	miR–31–5p, miR–345–3p, and miR–424–3p	RT–qPCR	SNORD60
Anu et al. [25]	2021	India	Case Control	49	50	99	AHSG	Discovery: Tandem Mass Tag (TMT)-based LC–MS relative quantification Validation: Parallel Reaction Monitoring (PRM)-targeted proteomics on Orbitrap	Proteomic: heavy isotope-labeled synthetic peptides spiked into each digest; quantification based on light/heavy transition area ratios and standard curves
							KRT6C		
							KLK1		
							BPIFB2		
							AZGP1		
Romani et al. [26]	2021	Italy	Prospective Cohort	83	53	136	miR–16–5p, miR–484, and miR–191–5p	RT–qPCR	Arithmetic mean of three reference miRNAs: miR–16–5p, miR–484, and miR–191–5p
He et al. [27]	2020	China	Case Control	49	14	63	miR–24–3p	microarray and RT–qPCR	cel–miR–39
Lee et al. [28]	2018	Taiwan	Case Control	41	24	65	Eotaxin	Human Cytokine/Chemokine Magnetic Bead Panel (MILLIPLEX MAP)	–
							IFN–y		
							IL-1β		
							IL-6		
							IL-8		
							MIP-1β		
							TNF-α		
Zhao et al. [29]	2018	China	Case Control	90	82	172	hsa_circ_0001874	Discovery: circRNA microarray Validation: RT–qPCR	β-actin
							hsa_circ_0001971		
							hsa_circ_0008068		
							hsa_circ_0000140		
							hsa_circ_0002632		
							hsa_circ_0008792		
							hsa_circ_0001874 + hsa_circ_0001971		
Gai et al. [30]	2018	Italy	Case Control	21	11	33	EV-miR-412-3p	RT–qPCR (miScript SYBR Green) on EV RNA	RNU6B and miR-191
							EV-miR-512-3p		
Awasthi et al. [31]	2017	India	Case Control	30	25	55	CYFRA 21-1	CYFRA 21-1, CA 19-9: ELISA kits	–
Wan et al. [32]	2017	Australia	Case Control	47	113	163	miR–9, miR–127, miR–134, miR–191, miR–222, and miR–455	RT–qPCR	SNORD96A
Duz et al. [33]	2016	Turkey	Case Control	24	25	49	miR–139–5p	microarray and RT–qPCR	Single RNU6B
Ghallab et al. [34]	2016	Egypt	Case Control	15	15	30	Chemerin	MMP-9: Quantikine ELISA kit, R&D Systems (DMP900)	–

OSCC, oral squamous cell carcinoma; RT–qPCR, reverse transcription quantitative polymerase chain reaction; NGS, next-generation sequencing; ELISA, enzyme-linked immunosorbent assay; LC–MS, liquid chromatography–mass spectrometry; PRM, parallel reaction monitoring; TMT, tandem mass tag; EV, extracellular vesicle; cfDNA, cell-free DNA; scfDNA, salivary cell-free DNA; circRNA, circular RNA; miRNA, microRNA; IFN-γ, interferon gamma; TNF-α, tumor necrosis factor alpha; IL, interleukin; MMP, matrix metalloproteinase; CYFRA 21-1, cytokeratin-19 fragment; AHSG, alpha-2-Heremans–Schmid glycoprotein; KLK1, kallikrein-1; KRT6C, keratin-6C; AZGP1, alpha-2-glycoprotein 1 zinc-binding; BPIFB2, BPI fold-containing family B member 2; SAT, spermidin e/spermine N^1^-acetyltransferase; OAZ1, ornithine decarboxylase antizyme 1; RLU, relative light units; NR, not reported; n, number of subjects.

**Table 2:** Baseline demographic and clinical characteristics of OSCC patients and control groups in the included studies. * indicates the training set; ** indicates the validation set.

Author	Gender				Age		Smoking Habit				Systemic Disease Excluded	Control Definition	Matching Reported
	OSCC		Control		OSCC (Mean ± SD or Median [IQR])	Control (Mean ± SD or Median [IQR])	OSCC		Control				
	Male (n, %)	Female (n, %)	Male (n, %)	Female (n, %)			Yes (n, %)	No (n, %)	Yes (n, %)	No (n, %)			
Kumar et al. [17]	37 (92.5)	3 (7.5)	29 (72.5)	11 (27.5)	47.6 ± 11.0	42.8 ± 8.3	–	–	–	–	Yes	Age-matched individuals with no oral pathological lesions on specialist examination	Age
Chu et al. [18]	191 (97)	5 (3)	167 (98)	4 (2)	Range 33–82, median 56	Range 30–82, median 59	172 (87.7)	24 (12.3)	165 (96.5)	6 (5)	Yes	Individuals above 30 years, with behaviors of smoking, betel nut chewing and/or alcohol consumption; no oral lesions; no personal history of other cancers or severe diseases	NR
Balakittnen et al. [19]	38 (76)	12 (24)	38 (63.3)	22 (36.7)	64.8 (47–87)	67.4 (43–89)	7 (14)	31 (62)	34 (56.7)	26 (43.3)	Yes	Clinically healthy individuals without OSCC or OPMD	NR
Garg et al. [20]	23 (76)	7 (24)	23 (76)	7 (24)	51.1 ± 12.75	38.5 ± 4.9	–	–	–	–	Yes	Age- and gender–matched healthy control individuals who had no smoking habit and had no significant oral or systemic disease	Age, sex, smoking habit
Tarrad et al. [21]	6 (50)	6 (50)	5 (41.7)	7 (58.3)	53.1 ± 8.0	38.7 ± 6.6	–	–	–	–	Yes	Systemically free individuals with no oral mucosal lesions, no systemic disease, no pregnancy/lactation, no current medication, and no clinical oral mucosal lesions on examination	Age, systemic disease
Rapado-González et al. [22]	11 (57.9)	8 (42.1)	–	–	72 (67–76)	68 (64–75)	–	–	–	–	NR	Healthy individuals with no visible oral lesions and no acute or chronic inflammatory conditions	Age, sex
Mehterov et al. [23]	30 (88.2)	4 (11.98)	–	–	60.9 (48–72)	–	32 (94.1)	2 (5.9)	–	–	NR	Individuals with no oral mucosal lesions	NR
Scholtz et al. [24]	28 (65)	15 (35)	16 (36)	28 (64)	57.9	57.6	24 (56)	12 (28)	6 (14)	21 (70)	NR	Individuals without diagnosis of OSCC	NR
Anu et al. [25]	–	–	–	–	54.6	54	28 (57.1)	21 (42.9)	35 (70)	15 (30)	No	Healthy volunteers without OSCC	Age, sex
Romani et al. [26]	43 (70) *	18 (30) *	28 (64) *	16 (36) *	66.7 (30–90) *	50.72 (22–92) *	36 (59) *	25 (41) *	21 (48) *	23 (52) *	NR	Individuals with no oral lesions	Smoking habit
	19 (68) **	9 (32) **	10 (71) **	4 (29) **	64.75 (24–91) **	75.57 (71–91) **	13 (46) **	14 (50) **	5 (35) **	4 (30) **			
He et al. [27]	30 (61.2)	19 (38.8)	8 (57.1)	6 (42.9)	–	–	20 (40.8)	29 (59.2)	5 (35.7)	9 (64.3)	Yes	Individuals with no oral mucosal lesions and no other malignant tumors or severe systemic diseases	Age, sex, smoking habit, systemic disease
Lee et al. [28]	36 (88)	5 (12)	20 (83)	4 (17)	–	–	9 (24)	15 (24)	32 (41)	9 (41)	Yes	Non-oral cancer patients with no history of malignancy and without osteomyelitis, HIV infection, active infection, immunodeficiency, autoimmune disease, or hepatitis	Age, sex
Zhao et al. [30]	53 (58.9)	37 (41.1)	–	–	–	–	42 (46.67)	48 (53.3)	–	–	NR	Individuals attending routine health check-up, with no oral diseases and no other cancerous diseases	Age, sex
Gai et al. [30]	12 (57)	9 (43)	7 (63.6)	4 (36.3)	65.75 (61–73)	61.64 (61.5–67.5)	6 (28.5)	15 (71.4)	3 (27.3)	11 (72.7)	Yes	Healthy subjects with no clinically detectable oral lesions, infections, or tumor history, matched for age, gender, and risk factors	Age, sex, risk factors
Awasthi et al. [31]	28 (93.3)	2 (6.7)	22 (88)	3 (12)	49.6 (25–70)	48.1 (25–68)	–	–	–	–	Yes	Age- and gender-matched healthy controls, without OSCC or premalignant lesions; chronic inflammatory diseases, autoimmune disorders, AIDS, prior malignancy or radiation exposure were excluded	Age, sex
Wan et al. [32]	83 (82.2)	19 (17.8)	59 (52.2)	54 (47.8)	61.9 ± 11.1	44.7 ± 11.4	84 (83.2)	17 (16.8)	42 (37.3)	71 (63.7)	NR	Individuals with no previous history of any malignancies in the head and neck areas	NR
Duz et al. [33]	19 (76)	6 (24)	21 (84)	4 (16)	54.08 ± 2.38	46.88 ± 3.63	–	–	–	–	Yes	Age- and gender-matched individuals without OSCC, without oral lesions and negative for hepatitis and HIV	Age, sex
Ghallab et al. [34]	6 (40)	9 (60)	8 (53.3)	7 (46.7)	43.26 ± 11.82	–	–	–	–	–	Yes	Healthy normal individuals: Free from systemic disease, inflammatory oral lesions, or periodontal disease, clinically healthy gingiva with zero plaque index, zero gingival index, zero clinical attachment loss, pocket depth ≤ 3 mm	Age, sex

OSCC oral squamous cell carcinoma; OPMD oral potentially malignant disorder; SD standard deviation; IQR interquartile range; NR not reported; n number of subjects; HIV human immunodeficiency virus.

**Table 3:** Summary of diagnostic performance metrics of the included studies.

Biomarker Type	Biomarker Evaluated_StudyID	TP	FP	TN	FN	Sensitivity (%)	Specificity (%)	AUC
Exosomal mRNAs	IL1_Kumar2025	27	11	29	13	67.5	71.8	0.71
	IL6_Kumar2025	32	13	27	8	80	67.5	0.79
	IL8_Kumar2025	24	9	31	16	60	77.5	0.69
	TNF-α_kumar2025	36	8	32	4	90	80	0.88
	S100_kumar2025	37	13	27	3	92.5	67.5	0.88
	SAT_kumar2025	31	9	31	9	77.5	77.5	0.82
	OAZ1_kumar2025	31	5	35	9	77.5	87.5	0.87
	MMP9_kumar2025	29	6	34	11	72.5	85	0.84
	Chemerin_kumar2025	32	11	29	8	80	71.8	0.78
Protein—Cytokine/inflammatory (signaling molecules; immune/inflammatory response)	Eotaxin_Lee2018	29	9	15	12	70.73	62.5	0.662
	IFN–y_Lee2018	33	12	12	8	80.49	50	0.657
	IL-1β_Lee2018	25	5	19	16	60.98	79.17	0.729
	IL-6_Lee2018	34	7	17	7	82.93	70.83	0.823
	IL-8_Lee2018	27	5	19	14	65.85	79.17	0.783
	MIP-1β_Lee2018	24	5	19	17	58.54	79.17	0.681
	TNF-α_Lee2018	16	0	24	25	39.02	100	0.749
	Chemerin_Ghallab2016	15	0	15	0	100	100	1
Protein—Non-cytokine structural/enzymatic/cancer-related proteins	MMP-1_Chu2024	169	57	27	350	86.22	86	0.902
	MMP–9_Ghallab2016	15	0	15	0	100	100	1
	AHSG_Anu2021	30	15	34	20	60	69.39	0.668
	KRT6C_Anu2021	30	18	31	20	60	63.26	0.64
	KLK1_Anu2021	29	15	34	21	58	69.39	0.66
	BPIFB2_Anu2021	35	17	32	15	70	64.58	0.66
	AZGP1_Anu2021	37	12	37	13	74	74.83	0.728
Protein—Structural/tumor-associated (cytoskeletal or tumor-related protein fragments)	CYFRA 21-1_Awashi2017	27	1	3	33	90	97	0.994
Nucleic acid—Cell-free DNA (cfDNA/scfDNA	scfDNA_ Rapado-González2022	16	4	3	11	83.33	73.33	0.821
Nucleic acid—miRNA panel	miRNA_Panel1_Balakittnen2024	43	7	6	54	86	90	0.95
	miRNA_Panel2_Sholtz2022	37	10	34	6	86	77	0.87
	miRNA_Panel3_Romani2021	71	8	45	12	85.4	85.1	0.923
	miRNA_Panel4_Wan2017	28	7	106	19	60	94	0.82
Nucleic acid—Single miRNA	miRNA–106a_Tarrad2023	12	3	0	9	100	70.8	0.9
	miRNA–21_Garg2023	24	9	21	6	80	70	0.89
	miRNA–184_Garg2023	24	8	22	6	80	74	0.87
	miR–30c–5p_Mehterov2021	43	11	31	7	86	74	0.82
	miR–24–3p_He2020	32	3	11	17	64.4	80	0.74
	miR–139–5p_Duz2016	18	4	21	7	75	84	0.81
Nucleic acid—circRNA Circular RNAs (circRNAs)	hsa_circ_0001874_Zhao2018	67	8	23	74	74.44	90.24	0.863
	hsa_circ_0001971_Zhao2018	68	10	22	72	75.56	87.8	0.845
	hsa_circ_0008068_Zhao2018	58	15	32	67	64.4	81.7	0.757
	hsa_circ_0000140_Zhao2018	64	15	26	67	71.1	81.7	0.803
	hsa_circ_0002632_Zhao2018	46	8	44	74	51.1	90.2	0.766
	hsa_circ_0008792_Zhao2018	39	12	51	70	43.3	85.4	0.658
	hsa_circ_0001874 + hsa_circ_0001971_Zhao2018	83	18	7	64	92.7	77.8	0.922
Nucleic acid—EV-associated miRNA	EV-miR-412-3p_Gai2018	16	1	5	10	75	90	0.847
	EV-miR-512-3p_Gai2018	18	3	3	8	86	75	0.871

TP true positive; FP false positive; TN true negative; FN false negative; AUC Area Under the Curve; EV extracellular vesicle; cfDNA cell-free DNA; scfDNA salivary cell-free DNA; circRNA circular RNA; hsa_circ Homo sapiens circular RNA; miRNA microRNA; IL interleukin; TNF-α tumor necrosis factor alpha; MMP matrix metalloproteinase; CYFRA 21-1 cytokeratin-19 fragment; AHSG alpha-2-Heremans–Schmid glycoprotein; KRT6C keratin-6C; KLK1 kallikrein-1; BPIFB2 BPI fold-containing family B member 2; AZGP1 alpha-2-glycoprotein 1 zinc-binding; SAT spermidine/spermine N^1^-acetyltransferase; OAZ1 ornithine decarboxylase antizyme-1.

**Table 4:** Predictive performance of salivary biomarkers across varied clinical prevalence scenarios based on Fagan’s nomograms.

Pre-Test Probability (Prevalence)	LR+	Post-Test Probability (Positive Result)	LR−	Post-Test Probability (Negative Result)	Clinical Context/Setting
0.01%	2	0%	0.47	0%	General population screening
0.5%	2	1%	0.47	0%	Low-risk/opportunistic screening
10%	2	21%	0.47	5%	Symptomatic/suspicion lesion clinic
40%	2	62%	0.47	24%	High-suspicion/referral setting

## Data Availability

The data supporting the findings of this study are contained within the article and its Supplementary Materials.

## Supplementary Materials

The following supporting information can be downloaded at: https://www.mdpi.com/article/10.3390/cancers18060970/s1, Table S1. Completed extracted data, Table S2. PRISMA 2020 Checklist, Figure S1. Subgroup analyses stratified by: (A) biomarker type, (B) single versus panel biomarkers, (C) detection platform, (D) geographic region, and (E) study quality. Reference [42] is cited in the Supplementary Materials.

## Abbreviations

The following abbreviations are used in this manuscript:

OSCC	Oral Squamous Cell Carcinoma
miRNA	microRNA
DOR	Diagnostic Odds Ratio
AUC	Area Under the Curve
SROC	Summary Receiver Operating Characteristic
CI	Confidence Interval
I^2^	Higgins’ I-squared statistic
lnDOR	Log Diagnostic Odds Ratio
RT–qPCR	Reverse Transcription Quantitative Polymerase Chain Reaction
EV	Extracellular Vesicle
cfDNA	Cell-Free DNA
NGS	Next-Generation Sequencing
PRISMA	Preferred Reporting Items for Systematic Reviews and Meta-Analyses
PROSPERO	International Prospective Register of Systematic Reviews
QUADAS-2	Quality Assessment of Diagnostic Accuracy Studies–2
TP	True Positive
FP	False Positive
FN	False Negative
TN	True Negative
