# Supplementary material for: Diagnostic Accuracy and Clinical Utility of Salivary Biomarkers in Oral Squamous Cell Carcinoma: A Meta-Analysis
Source: Cancers (Basel). 2026 Mar 17;18(6):970. doi: 10.3390/cancers18060970 (PMC13025114; doi:10.3390/cancers18060970)
Supplement: Supplementary file 1 [file cancers-18-00970-s001.zip › cancers-4165410-supplementary.pdf]

Supplementary Materials

Table S1. Completed extracted data

studyid	tp	fp	fn	tn
IL1_Kumar2025	27	11	13	29
IL6_Kumar2025	32	13	8	27
IL8_Kumar2025	24	9	16	31
TNF-α_kumar2025	36	8	4	32
S100_kumar2025	37	13	3	27
SAT_kumar2025	31	9	9	31
OAZ1_kumar2025	31	5	9	35
MMP9_kumar2025	29	6	11	34
Chemerin_kumar2025	32	11	29	8
MMP-1_Chu2024	169	57	350	27
miRNAPanel1_Balakittnen2024	43	7	54	6
miRNA–21_Garg2023	24	9	6	21
miRNA–184_Garg2023	24	8	6	22
miRNA–106a_Tarrad2023	12	3	9	0
scfDNA_Gonzalez2022	16	4	11	3
miRNAPanel2_Sholtz2022	37	10	6	34
miR–30c–5p_Mehterov2022	43	11	7	31
AHSG_Anu2021	30	15	20	34
KRT6C_Anu2021	30	18	20	31
KLK1_Anu2021	29	15	21	34
BPIFB2_Anu2021	35	17	15	32
AZGP1_Anu2021	37	12	13	37
miRNAPanel3_Romani2021	71	8	45	12
miR–24–3p_He2020	32	3	17	11
Eotaxin_Lee2018	29	9	12	15
IFN–y_Lee2018	33	12	8	12
IL-1β_Lee2018	25	5	16	19
IL-6_Lee2018	34	7	7	17
IL-8_Lee2018	27	5	14	19
MIP-1β_Lee2018	24	5	17	19
TNF-α_Lee2018	16	0	25	24
hsa_circ_0001874_Zhao2018	67	8	74	23
hsa_circ_0001971_Zhao2018	68	10	72	22
hsa_circ_0008068_Zhao2018	58	15	67	32
hsa_circ_0000140_Zhao2018	64	15	67	26
hsa_circ_0002632_Zhao2018	46	8	74	44
hsa_circ_0008792_Zhao2018	39	12	70	51
hsa_circ_0001874 +_Zhao2018	83	18	64	7
EV-miR-412-3p_Gai2018	16	1	10	5

EV-miR-512-3p_Gai2018	18	3	8	3
CYFRA 21-1_Awashi2017	27	1	33	3
miRNAPanel4_Wan2017	28	7	19	106
miR–139–5p_Duz2016	18	4	7	21
Chemerin_Ghallab2016	15	0	0	15
MMP–9_Ghallab2016	15	0	0	15

Table S2. PRISMA 2020 Checklist.

Section and Topic	Item #	Checklist item	Location where item is reported
**TITLE**			
Title	1	Identify the report as a systematic review.	Page 1
**ABSTRACT**			
Abstract	2	See the PRISMA 2020 for Abstracts checklist.	Page 2
**INTRODUCTION**			
Rationale	3	Describe the rationale for the review in the context of existing knowledge.	Page 3
Objectives	4	Provide an explicit statement of the objective(s) or question(s) the review addresses.	Page 3
**METHODS**			
Eligibility criteria	5	Specify the inclusion and exclusion criteria for the review and how studies were grouped for the syntheses.	Page 4
Information sources	6	Specify all databases, registers, websites, organisations, reference lists and other sources searched or consulted to identify studies. Specify the date when each source was last searched or consulted.	Page 3
Search strategy	7	Present the full search strategies for all databases, registers and websites, including any filters and limits used.	Page 3-4
Selection process	8	Specify the methods used to decide whether a study met the inclusion criteria of the review, including how many reviewers screened each record and each report retrieved, whether they worked independently, and if applicable, details of automation tools used in the process.	Page 3-4
Data collection process	9	Specify the methods used to collect data from reports, including how many reviewers collected data from each report, whether they worked independently, any processes for obtaining or confirming data from study investigators, and if applicable, details of automation tools used in the process.	Page 4
Data items	10a	List and define all outcomes for which data were sought. Specify whether all results that were compatible with each outcome domain in each study were sought (e.g. for all measures, time points, analyses), and if not, the methods used to decide which results to collect.	Page 4
	10b	List and define all other variables for which data were sought (e.g. participant and intervention characteristics, funding sources). Describe any assumptions made about any missing or unclear information.	Page 4
Study risk of bias assessment	11	Specify the methods used to assess risk of bias in the included studies, including details of the tool(s) used, how many reviewers assessed each study and whether they worked independently, and if applicable, details of automation tools used in the process.	Page 4
Effect measures	12	Specify for each outcome the effect measure(s) (e.g. risk ratio, mean difference) used in the synthesis or presentation of results.	Page 5
Synthesis methods	13a	Describe the processes used to decide which studies were eligible for each synthesis (e.g. tabulating the study intervention characteristics and comparing against the planned groups for each synthesis (item #5)).	Page 4
	13b	Describe any methods required to prepare the data for presentation or synthesis, such as handling of missing summary statistics, or data conversions.	Page 4
	13c	Describe any methods used to tabulate or visually display results of individual studies and syntheses.	Page 4
	13d	Describe any methods used to synthesize results and provide a rationale for the choice(s). If meta-analysis was performed, describe the model(s), method(s) to identify the presence and extent of statistical heterogeneity, and software package(s) used.	Page 4
	13e	Describe any methods used to explore possible causes of heterogeneity among study results (e.g. subgroup analysis, meta-regression).	Page 5
	13f	Describe any sensitivity analyses conducted to assess robustness of the synthesized results.	Page 5
Reporting bias	14	Describe any methods used to assess risk of bias due to missing results in a synthesis (arising from reporting biases).	Page 4

Section and Topic	Item #	Checklist item	Location where item is reported
assessment			
Certainty assessment	15	Describe any methods used to assess certainty (or confidence) in the body of evidence for an outcome.	Page 4
**RESULTS**			
Study selection	16a	Describe the results of the search and selection process, from the number of records identified in the search to the number of studies included in the review, ideally using a flow diagram.	Page 5
	16b	Cite studies that might appear to meet the inclusion criteria, but which were excluded, and explain why they were excluded.	Page 5
Study characteristics	17	Cite each included study and present its characteristics.	Page 5
Risk of bias in studies	18	Present assessments of risk of bias for each included study.	Page 7
Results of individual studies	19	For all outcomes, present, for each study: (a) summary statistics for each group (where appropriate) and (b) an effect estimate and its precision (e.g. confidence/credible interval), ideally using structured tables or plots.	Page 4 & supplementary materials
Results of syntheses	20a	For each synthesis, briefly summarise the characteristics and risk of bias among contributing studies.	Page 7
	20b	Present results of all statistical syntheses conducted. If meta-analysis was done, present for each the summary estimate and its precision (e.g. confidence/credible interval) and measures of statistical heterogeneity. If comparing groups, describe the direction of the effect.	Page 6-7
	20c	Present results of all investigations of possible causes of heterogeneity among study results.	Page 6-7
	20d	Present results of all sensitivity analyses conducted to assess the robustness of the synthesized results.	Page 6-7
Reporting biases	21	Present assessments of risk of bias due to missing results (arising from reporting biases) for each synthesis assessed.	Page 6
Certainty of evidence	22	Present assessments of certainty (or confidence) in the body of evidence for each outcome assessed.	Page 6-7
**DISCUSSION**			
Discussion	23a	Provide a general interpretation of the results in the context of other evidence.	Page 7
	23b	Discuss any limitations of the evidence included in the review.	Page 8
	23c	Discuss any limitations of the review processes used.	Page 8
	23d	Discuss implications of the results for practice, policy, and future research.	Page 8
**OTHER INFORMATION**			
Registration and protocol	24a	Provide registration information for the review, including register name and registration number, or state that the review was not registered.	Page 3
	24b	Indicate where the review protocol can be accessed, or state that a protocol was not prepared.	Page 4
	24c	Describe and explain any amendments to information provided at registration or in the protocol.	Page 9
Support	25	Describe sources of financial or non-financial support for the review, and the role of the funders or sponsors in the review.	Page 9
Competing interests	26	Declare any competing interests of review authors.	Page 9
Availability of data, code and	27	Report which of the following are publicly available and where they can be found: template data collection forms; data extracted from included studies; data used for all analyses; analytic code; any other materials used in the review.	Page 9

Section and Topic	Item #	Checklist item	Location where item is reported
other materials			

From: Page MJ, McKenzie JE, Bossuyt PM, Boutron I, Hoffmann TC, Mulrow CD, et al. The PRISMA 2020 statement: an updated guideline for reporting systematic reviews. BMJ 2021;372:n71. doi: 10.1136/bmj.n71. This work is licensed under CC BY 4.0. To view a copy of this license, visit https://creativecommons.org/licenses/by/4.0/

Figure S1. Subgroup analyses stratified by: (A) biomarker type, (B) single versus panel biomarkers, (C) detection platform, (D) geographic region, and (E) study quality.

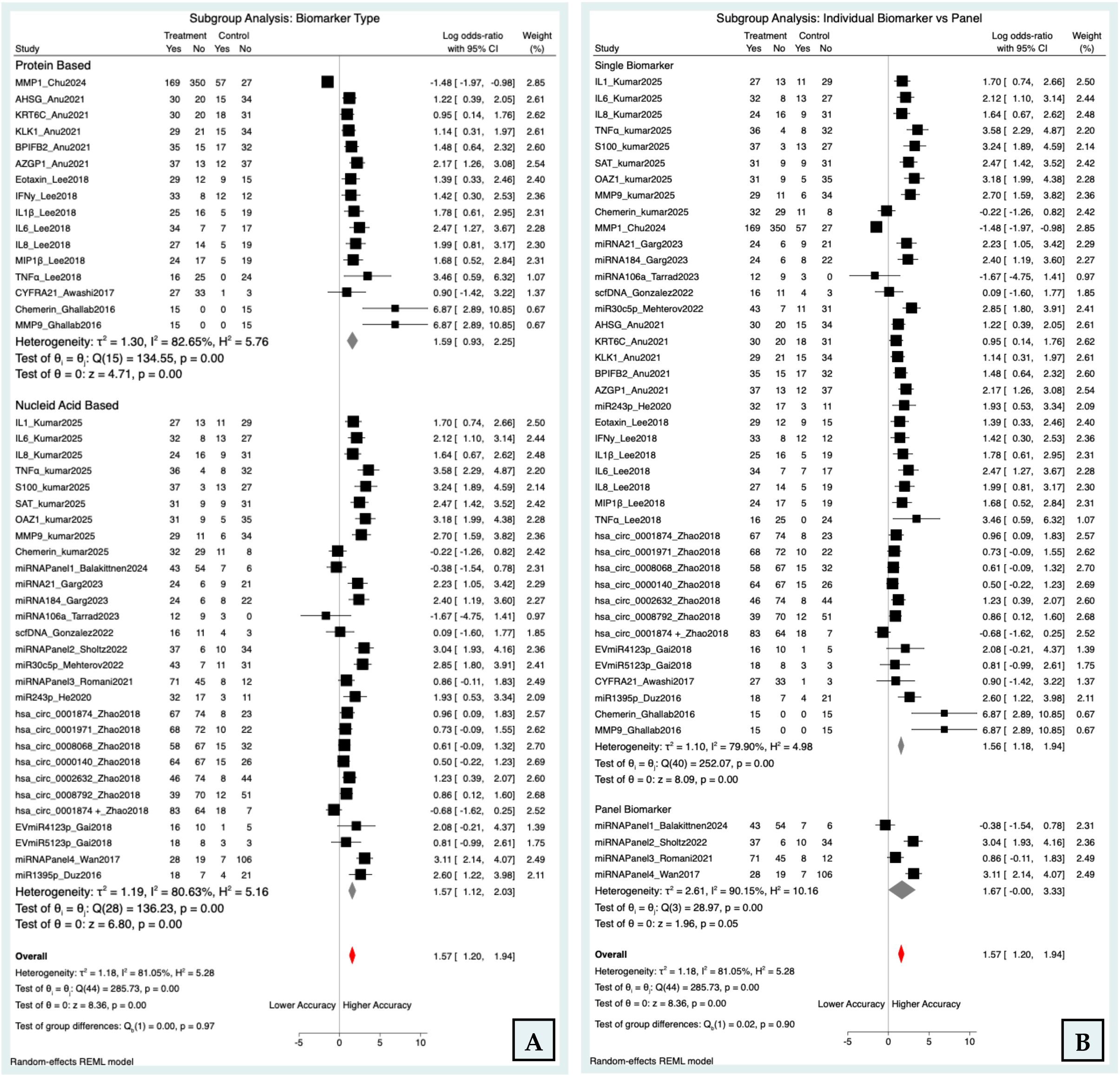

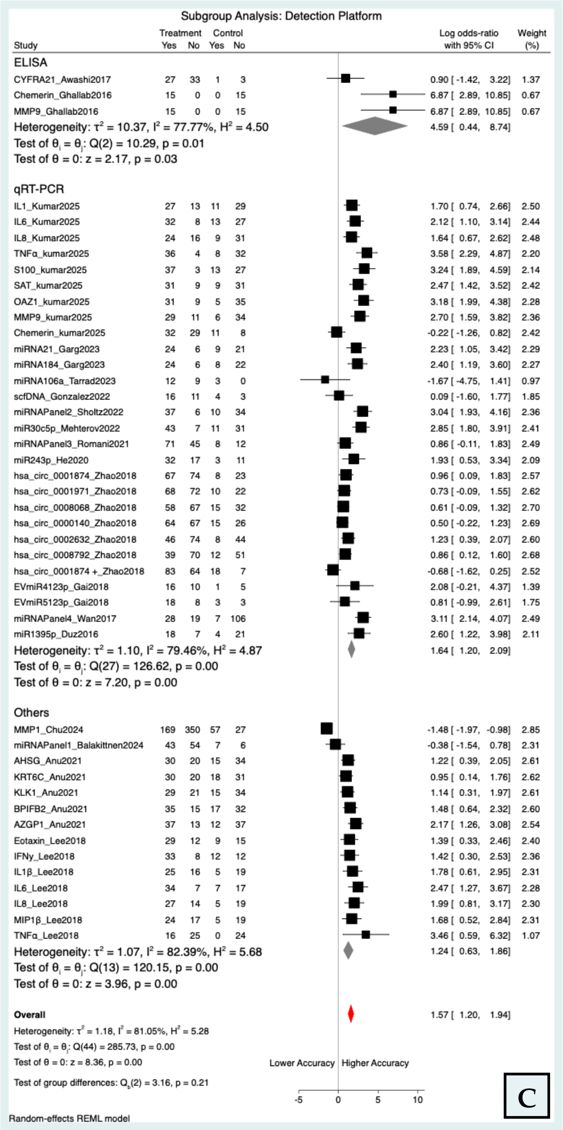
Subgroup Analysis: Detection Platform
Study | Treatment Yes No | Control Yes No | Log odds-ratio with 95% CI | Weight (%)
ELISA
CYFRA21_Awashi2017 27 33 1 3 0.90 [-1.42, 3.22] 1.37
Chemerin_Ghallab2016 15 0 0 15 6.87 [2.89, 10.85] 0.67
MMP9_Ghallab2016 15 0 0 15 6.87 [2.89, 10.85] 0.67
Heterogeneity: τ² = 10.37, I² = 77.77%, H² = 4.50 4.59 [0.44, 8.74]
Test of θi = θj: Q(2) = 10.29, p = 0.01
Test of θ = 0: z = 2.17, p = 0.03
qRT-PCR
IL1_Kumar2025 27 13 11 29 1.70 [0.74, 2.66] 2.50
IL6_Kumar2025 32 8 13 27 2.12 [1.10, 3.14] 2.44
IL8_Kumar2025 24 16 9 31 1.64 [0.67, 2.62] 2.48
TNFα_kumar2025 36 4 8 32 3.58 [2.29, 4.87] 2.20
S100_kumar2025 37 3 13 27 3.24 [1.89, 4.59] 2.14
SAT_kumar2025 31 9 9 31 2.47 [1.42, 3.52] 2.42
OAZ1_kumar2025 31 9 5 35 3.18 [1.99, 4.38] 2.28
MMP9_kumar2025 29 11 6 34 2.70 [1.59, 3.82] 2.36
Chemerin_kumar2025 32 29 11 8 -0.22 [-1.26, 0.82] 2.42
miRNA21_Garg2023 24 6 9 21 2.23 [1.05, 3.42] 2.29
miRNA184_Garg2023 24 6 8 22 2.40 [1.19, 3.60] 2.27
miRNA106a_Tarrad2023 12 9 3 0 -1.67 [-4.75, 1.41] 0.97
scfDNA_Gonzalez2022 16 11 4 3 0.09 [-1.60, 1.77] 1.85
miRNAPanel2_Sholtz2022 37 6 10 34 3.04 [1.93, 4.16] 2.36
miR30c5p_Mehterov2022 43 7 11 31 2.85 [1.80, 3.91] 2.41
miRNAPanel3_Romani2021 71 45 8 12 0.86 [-0.11, 1.83] 2.49
miR243p_He2020 32 17 3 11 1.93 [0.53, 3.34] 2.09
hsa_circ_0001874_Zhao2018 67 74 8 23 0.96 [0.09, 1.83] 2.57
hsa_circ_0001971_Zhao2018 68 72 10 22 0.73 [-0.09, 1.55] 2.62
hsa_circ_0008068_Zhao2018 58 67 15 32 0.61 [-0.09, 1.32] 2.70
hsa_circ_0000140_Zhao2018 64 67 15 26 0.50 [-0.22, 1.23] 2.69
hsa_circ_0002632_Zhao2018 46 74 8 44 1.23 [0.39, 2.07] 2.60
hsa_circ_0008792_Zhao2018 39 70 12 51 0.86 [0.12, 1.60] 2.68
hsa_circ_0001874 +_Zhao2018 83 64 18 7 -0.68 [-1.62, 0.25] 2.52
EVmiR4123p_Gai2018 16 10 1 5 2.08 [-0.21, 4.37] 1.39
EVmiR5123p_Gai2018 18 8 3 3 0.81 [-0.99, 2.61] 1.75
miRNAPanel4_Wan2017 28 19 7 106 3.11 [2.14, 4.07] 2.49
miR1395p_Duz2016 18 7 4 21 2.60 [1.22, 3.98] 2.11
Heterogeneity: τ² = 1.10, I² = 79.46%, H² = 4.87 1.64 [1.20, 2.09]
Test of θi = θj: Q(27) = 126.62, p = 0.00
Test of θ = 0: z = 7.20, p = 0.00
Others
MMP1_Chu2024 169 350 57 27 -1.48 [-1.97, -0.98] 2.85
miRNAPanel1_Balakittnen2024 43 54 7 6 -0.38 [-1.54, 0.78] 2.31
AHSG_Anu2021 30 20 15 34 1.22 [0.39, 2.05] 2.61
KRT6C_Anu2021 30 20 18 31 0.95 [0.14, 1.76] 2.62
KLK1_Anu2021 29 21 15 34 1.14 [0.31, 1.97] 2.61
BPIFB2_Anu2021 35 15 17 32 1.48 [0.64, 2.32] 2.60
AZGP1_Anu2021 37 13 12 37 2.17 [1.26, 3.08] 2.54
Eotaxin_Lee2018 29 12 9 15 1.39 [0.33, 2.46] 2.40
IFNγ_Lee2018 33 8 12 12 1.42 [0.30, 2.53] 2.36
IL1β_Lee2018 25 16 5 19 1.78 [0.61, 2.95] 2.31
IL6_Lee2018 34 7 7 17 2.47 [1.27, 3.67] 2.28
IL8_Lee2018 27 14 5 19 1.99 [0.81, 3.17] 2.30
MIP1β_Lee2018 24 17 5 19 1.68 [0.52, 2.84] 2.31
TNFα_Lee2018 16 25 0 24 3.46 [0.59, 6.32] 1.07
Heterogeneity: τ² = 1.07, I² = 82.39%, H² = 5.68 1.24 [0.63, 1.86]
Test of θi = θj: Q(13) = 120.15, p = 0.00
Test of θ = 0: z = 3.96, p = 0.00
Overall 1.57 [1.20, 1.94]
Heterogeneity: τ² = 1.18, I² = 81.05%, H² = 5.28
Test of θi = θj: Q(44) = 285.73, p = 0.00
Test of θ = 0: z = 8.36, p = 0.00
Test of group differences: Qb(2) = 3.16, p = 0.21
Lower Accuracy
Higher Accuracy
-5 0 5 10
Random-effects REML model
C

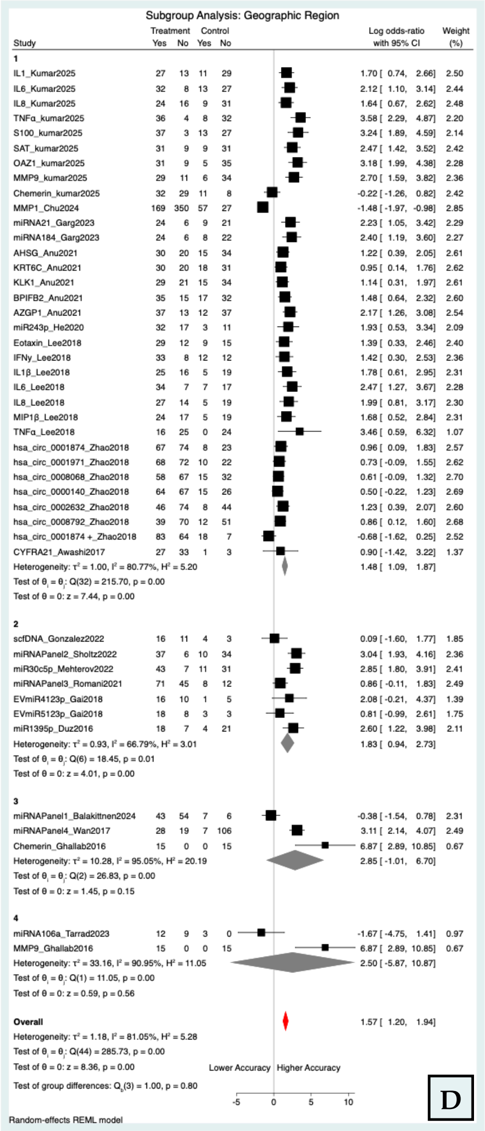
Subgroup Analysis: Geographic Region
Study | Treatment Yes No | Control Yes No | Log odds-ratio with 95% CI | Weight (%)
1
IL1_Kumar2025 27 13 11 29 1.70 [0.74, 2.66] 2.50
IL6_Kumar2025 32 8 13 27 2.12 [1.10, 3.14] 2.44
IL8_Kumar2025 24 16 9 31 1.64 [0.67, 2.62] 2.48
TNFα_kumar2025 36 4 8 32 3.58 [2.29, 4.87] 2.20
S100_kumar2025 37 3 13 27 3.24 [1.89, 4.59] 2.14
SAT_kumar2025 31 9 9 31 2.47 [1.42, 3.52] 2.42
OAZ1_kumar2025 31 9 5 35 3.18 [1.99, 4.38] 2.28
MMP9_kumar2025 29 11 6 34 2.70 [1.59, 3.82] 2.36
Chemerin_kumar2025 32 29 11 8 -0.22 [-1.26, 0.82] 2.42
MMP1_Chu2024 169 350 57 27 -1.48 [-1.97, -0.98] 2.85
miRNA21_Garg2023 24 6 9 21 2.23 [1.05, 3.42] 2.29
miRNA184_Garg2023 24 6 8 22 2.40 [1.19, 3.60] 2.27
AHSG_Anu2021 30 20 15 34 1.22 [0.39, 2.05] 2.61
KRT6C_Anu2021 30 20 18 31 0.95 [0.14, 1.76] 2.62
KLK1_Anu2021 29 21 15 34 1.14 [0.31, 1.97] 2.61
BPIFB2_Anu2021 35 15 17 32 1.48 [0.64, 2.32] 2.60
AZGP1_Anu2021 37 13 12 37 2.17 [1.26, 3.08] 2.54
miR243p_He2020 32 17 3 11 1.93 [0.53, 3.34] 2.09
Eotaxin_Lee2018 29 12 9 15 1.39 [0.33, 2.46] 2.40
IFNγ_Lee2018 33 8 12 12 1.42 [0.30, 2.53] 2.36
IL1β_Lee2018 25 16 5 19 1.78 [0.61, 2.95] 2.31
IL6_Lee2018 34 7 7 17 2.47 [1.27, 3.67] 2.28
IL8_Lee2018 27 14 5 19 1.99 [0.81, 3.17] 2.30
MIP1β_Lee2018 24 17 5 19 1.68 [0.52, 2.84] 2.31
TNFα_Lee2018 16 25 0 24 3.46 [0.59, 6.32] 1.07
hsa_circ_0001874_Zhao2018 67 74 8 23 0.96 [0.09, 1.83] 2.57
hsa_circ_0001971_Zhao2018 68 72 10 22 0.73 [-0.09, 1.55] 2.62
hsa_circ_0008068_Zhao2018 58 67 15 32 0.61 [-0.09, 1.32] 2.70
hsa_circ_0000140_Zhao2018 64 67 15 26 0.50 [-0.22, 1.23] 2.69
hsa_circ_0002632_Zhao2018 46 74 8 44 1.23 [0.39, 2.07] 2.60
hsa_circ_0008792_Zhao2018 39 70 12 51 0.86 [0.12, 1.60] 2.68
hsa_circ_0001874 +_Zhao2018 83 64 18 7 -0.68 [-1.62, 0.25] 2.52
CYFRA21_Awashi2017 27 33 1 3 0.90 [-1.42, 3.22] 1.37
Heterogeneity: τ² = 1.00, I² = 80.77%, H² = 5.20 1.48 [1.09, 1.87]
Test of θi = θj: Q(32) = 215.70, p = 0.00
Test of θ = 0: z = 7.44, p = 0.00
2
scfDNA_Gonzalez2022 16 11 4 3 0.09 [-1.60, 1.77] 1.85
miRNAPanel2_Sholtz2022 37 6 10 34 3.04 [1.93, 4.16] 2.36
miR30c5p_Mehterov2022 43 7 11 31 2.85 [1.80, 3.91] 2.41
miRNAPanel3_Romani2021 71 45 8 12 0.86 [-0.11, 1.83] 2.49
EVmiR4123p_Gai2018 16 10 1 5 2.08 [-0.21, 4.37] 1.39
EVmiR5123p_Gai2018 18 8 3 3 0.81 [-0.99, 2.61] 1.75
miR1395p_Duz2016 18 7 4 21 2.60 [1.22, 3.98] 2.11
Heterogeneity: τ² = 0.93, I² = 66.79%, H² = 3.01 1.83 [0.94, 2.73]
Test of θi = θj: Q(6) = 18.45, p = 0.01
Test of θ = 0: z = 4.01, p = 0.00
3
miRNAPanel1_Balakittnen2024 43 54 7 6 -0.38 [-1.54, 0.78] 2.31
miRNAPanel4_Wan2017 28 19 7 106 3.11 [2.14, 4.07] 2.49
Chemerin_Ghallab2016 15 0 0 15 6.87 [2.89, 10.85] 0.67
Heterogeneity: τ² = 10.28, I² = 95.05%, H² = 20.19 2.85 [-1.01, 6.70]
Test of θi = θj: Q(2) = 26.83, p = 0.00
Test of θ = 0: z = 1.45, p = 0.15
4
miRNA106a_Tarrad2023 12 9 3 0 -1.67 [-4.75, 1.41] 0.97
MMP9_Ghallab2016 15 0 0 15 6.87 [2.89, 10.85] 0.67
Heterogeneity: τ² = 33.16, I² = 90.95%, H² = 11.05 2.50 [-5.87, 10.87]
Test of θi = θj: Q(1) = 11.05, p = 0.00
Test of θ = 0: z = 0.59, p = 0.56
Overall 1.57 [1.20, 1.94]
Heterogeneity: τ² = 1.18, I² = 81.05%, H² = 5.28
Test of θi = θj: Q(44) = 285.73, p = 0.00
Test of θ = 0: z = 8.36, p = 0.00
Test of group differences: Qb(3) = 1.00, p = 0.80
Lower Accuracy
Higher Accuracy
-5 0 5 10
Random-effects REML model
D

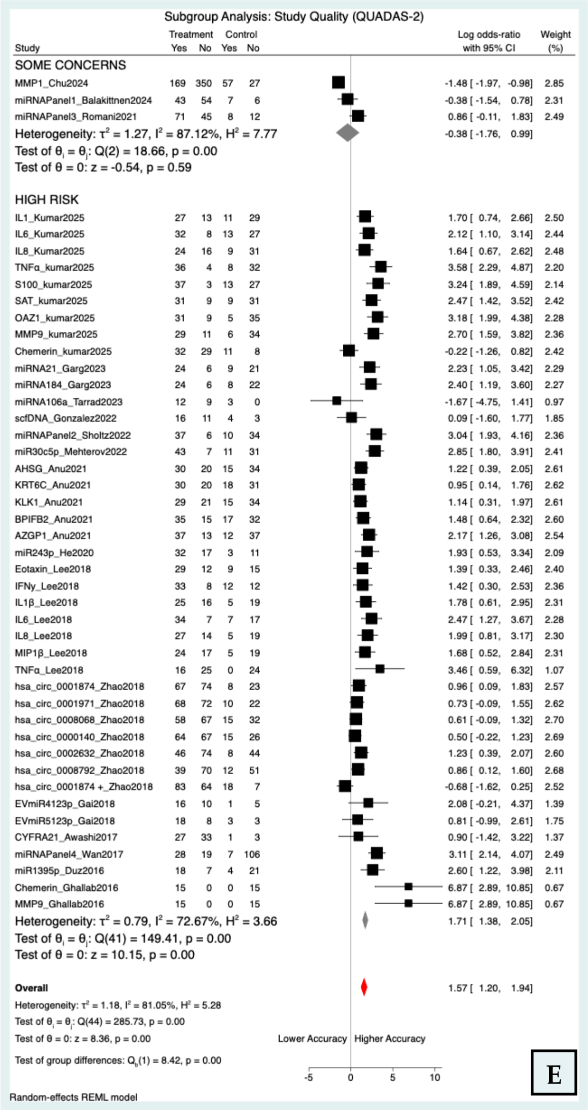

Subgroup Analysis: Study Quality (QUADAS-2)
Study | Treatment Yes | Treatment No | Control Yes | Control No | Log odds-ratio with 95% CI | Weight (%)
SOME CONCERNS
MMP1_Chu2024 169 350 57 27 -1.48 [-1.97, -0.98] 2.85
miRNAPanel1_Balakittnen2024 43 54 7 6 -0.38 [-1.54, 0.78] 2.31
miRNAPanel3_Romani2021 71 45 8 12 0.86 [-0.11, 1.83] 2.49
Heterogeneity: τ² = 1.27, I² = 87.12%, H² = 7.77 -0.38 [-1.76, 0.99]
Test of θi = θj: Q(2) = 18.66, p = 0.00
Test of θ = 0: z = -0.54, p = 0.59
HIGH RISK
IL1_Kumar2025 27 13 11 29 1.70 [0.74, 2.66] 2.50
IL6_Kumar2025 32 8 13 27 2.12 [1.10, 3.14] 2.44
IL8_Kumar2025 24 16 9 31 1.64 [0.67, 2.62] 2.48
TNFα_kumar2025 36 4 8 32 3.58 [2.29, 4.87] 2.20
S100_kumar2025 37 3 13 27 3.24 [1.89, 4.59] 2.14
SAT_kumar2025 31 9 9 31 2.47 [1.42, 3.52] 2.42
OAZ1_kumar2025 31 9 5 35 3.18 [1.99, 4.38] 2.28
MMP9_kumar2025 29 11 6 34 2.70 [1.59, 3.82] 2.36
Chemerin_kumar2025 32 29 11 8 -0.22 [-1.26, 0.82] 2.42
miRNA21_Garg2023 24 6 9 21 2.23 [1.05, 3.42] 2.29
miRNA184_Garg2023 24 6 8 22 2.40 [1.19, 3.60] 2.27
miRNA106a_Tarrad2023 12 9 3 0 -1.67 [-4.75, 1.41] 0.97
scfDNA_Gonzalez2022 16 11 4 3 0.09 [-1.60, 1.77] 1.85
miRNAPanel2_Sholtz2022 37 6 10 34 3.04 [1.93, 4.16] 2.36
miR30c5p_Mehterov2022 43 7 11 31 2.85 [1.80, 3.91] 2.41
AHSG_Anu2021 30 20 15 34 1.22 [0.39, 2.05] 2.61
KRT6C_Anu2021 30 20 18 31 0.95 [0.14, 1.76] 2.62
KLK1_Anu2021 29 21 15 34 1.14 [0.31, 1.97] 2.61
BPIFB2_Anu2021 35 15 17 32 1.48 [0.64, 2.32] 2.60
AZGP1_Anu2021 37 13 12 37 2.17 [1.26, 3.08] 2.54
miR243p_He2020 32 17 3 11 1.93 [0.53, 3.34] 2.09
Eotaxin_Lee2018 29 12 9 15 1.39 [0.33, 2.46] 2.40
IFNy_Lee2018 33 8 12 12 1.42 [0.30, 2.53] 2.36
IL1β_Lee2018 25 16 5 19 1.78 [0.61, 2.95] 2.31
IL6_Lee2018 34 7 7 17 2.47 [1.27, 3.67] 2.28
IL8_Lee2018 27 14 5 19 1.99 [0.81, 3.17] 2.30
MIP1β_Lee2018 24 17 5 19 1.68 [0.52, 2.84] 2.31
TNFα_Lee2018 16 25 0 24 3.46 [0.59, 6.32] 1.07
hsa_circ_0001874_Zhao2018 67 74 8 23 0.96 [0.09, 1.83] 2.57
hsa_circ_0001971_Zhao2018 68 72 10 22 0.73 [-0.09, 1.55] 2.62
hsa_circ_0008068_Zhao2018 58 67 15 32 0.61 [-0.09, 1.32] 2.70
hsa_circ_0000140_Zhao2018 64 67 15 26 0.50 [-0.22, 1.23] 2.69
hsa_circ_0002632_Zhao2018 46 74 8 44 1.23 [0.39, 2.07] 2.60
hsa_circ_0008792_Zhao2018 39 70 12 51 0.86 [0.12, 1.60] 2.68
hsa_circ_0001874 +_Zhao2018 83 64 18 7 -0.68 [-1.62, 0.25] 2.52
EVmiR4123p_Gai2018 16 10 1 5 2.08 [-0.21, 4.37] 1.39
EVmiR5123p_Gai2018 18 8 3 3 0.81 [-0.99, 2.61] 1.75
CYFRA21_Awashi2017 27 33 1 3 0.90 [-1.42, 3.22] 1.37
miRNAPanel4_Wan2017 28 19 7 106 3.11 [2.14, 4.07] 2.49
miR1395p_Duz2016 18 7 4 21 2.60 [1.22, 3.98] 2.11
Chemerin_Ghallab2016 15 0 0 15 6.87 [2.89, 10.85] 0.67
MMP9_Ghallab2016 15 0 0 15 6.87 [2.89, 10.85] 0.67
Heterogeneity: τ² = 0.79, I² = 72.67%, H² = 3.66 1.71 [1.38, 2.05]
Test of θi = θj: Q(41) = 149.41, p = 0.00
Test of θ = 0: z = 10.15, p = 0.00
Overall 1.57 [1.20, 1.94]
Heterogeneity: τ² = 1.18, I² = 81.05%, H² = 5.28
Test of θi = θj: Q(44) = 285.73, p = 0.00
Test of θ = 0: z = 8.36, p = 0.00
Lower Accuracy
Higher Accuracy
Test of group differences: Qb(1) = 8.42, p = 0.00
-5
0
5
10
E
Random-effects REML model
